# Craniofacial and Radiological Features as Diagnostic Clues to Unmask Acromegaly: A Case Report

**DOI:** 10.7759/cureus.100144

**Published:** 2025-12-26

**Authors:** Jhalak Modi, Eiman Ibrahim, Katherine LaFoe, Tushar Ralhan, Esra Demirtas

**Affiliations:** 1 Internal Medicine, University of Missouri, Columbia, USA; 2 Endocrinology, University of Missouri, Columbia, USA; 3 Radiology, University of Missouri, Columbia, USA; 4 Endocrinology and Diabetes, University of Missouri, Columbia, USA

**Keywords:** acromegaly comorbidities, acromegaly symptoms, awareness of cardiovascular disease, internal medicine and endocrinology, pituitary adenoma size, pituitary disorders, pulmonary disease

## Abstract

A 70-year-old female with a past medical history of type 2 diabetes mellitus (treated with insulin), hypertension, and bipolar disorder was admitted to the Intensive Care Unit after suffering cardiac arrest post-seizure. During the initial workup, CT of the head was notable for marked, diffuse calvarial thickening (~17 mm), a large sella turcica, and no evidence of pituitary apoplexy. MRI of the head showed a 1.9 cm pituitary macroadenoma. The physical examination was notable for thick facial features such as macroglossia, acrochordon, and fleshy hands - consistent with acromegaly. Insulin-like growth factor-1 (IGF-1) was elevated at 570 ng/mL (Z score: 4; normal 34-245). IGF-binding protein 3 (IGFBP-3) was elevated at 9 µg/mL (normal 3-6). However, the patient had never previously been diagnosed with acromegaly.

Throughout the course of her admission, the patient required multiple bronchoscopies due to thick secretions and mucus plugging. The etiology of her respiratory failure and sudden cardiac arrest remained undetermined after a comprehensive diagnostic workup. While acromegaly typically manifests with musculoskeletal changes and endocrine abnormalities - including diabetes mellitus, hypertension, and galactorrhea - it also carries a substantial risk of secondary respiratory and cardiac complications. This report highlights the importance of early diagnosis of acromegaly, specifically in identifying, being cognizant of, and preventing respiratory and cardiac complications.

## Introduction

Acromegaly is characterized by high levels of growth hormone (GH), which in turn leads to the secretion of insulin-like growth factor-1 (IGF-1), with a prevalence of 4,600 per million. Dineen et al. (2017) suggest that acromegaly proves to be a challenging diagnosis, in that there are clinical manifestations such as macroglossia, coarse facial features, macrognathia, as well as large hands and feet. Common manifestations of acromegaly include diabetes mellitus, hypertension, and obstructive sleep apnea (OSA). Oftentimes, the cause of acromegaly is a growth in the pituitary gland, as this is where hormones are produced in the body. As these manifestations are common to a variety of metabolic and cardiovascular disorders, they may mask the underlying diagnosis of acromegaly, particularly in patients in whom classical phenotypic features are not pronounced [1]. 

The mortality rate in patients with untreated acromegaly is two to four times higher than in the general population. Cardiovascular disease has typically been reported to be the main cause of death in these patients, contributing to nearly 50% of mortality. Nevertheless, malignancy is now considered to be the most common cause of mortality in acromegaly, according to recent studies such as that by Arosio et al. (2024) [2]. There is a direct association between increased mortality rates and excess GH and IGF-1 secretion [3]. Achieving normalization of GH and IGF-1 has been shown to lower both mortality and disease-related morbidity. Additionally, higher mortality rates are positively correlated with acromegaly patients who have colorectal cancer. However, advancements in the management of acromegaly have led to significant improvements in survival rates and quality of life [4].

This case poses the question of larger-scale complications of acromegaly - such as acute-on-chronic respiratory failure and cardiac arrest. Since this patient was not diagnosed with acromegaly until postmortem, it is challenging to say whether preventative measures throughout her lifetime to reduce her cardiac risk would have played a large role in the end of her life. Awareness should be raised among clinicians and patients regarding the elevated risk of cardiac hypertrophy and abnormal pulmonary growth associated with acromegaly, which can lead to both obstructive and restrictive ventilatory defects.

## Case presentation

At the age of 61, the patient began having headaches, vision loss, and right-sided numbness. She has a history of infrarenal abdominal aortic aneurysm, which was repaired in 2017. She also had a history of three transient ischemic attacks, which all occurred during her mid-50s. She was not on a statin or aspirin at the age of 61. She saw neurology and vascular surgery due to concerns about an aneurysm in her right carotid artery, but no aneurysm repair was performed. Her MRI of the brain in 2020 did not note any pituitary adenoma at the time. 

Upon her admission to the ICU in 2025, post-seizure and post-cardiac arrest, her lab findings were concerning for a urinary tract infection. No other source of infection had been discovered. She was placed on antibiotics for her urinary tract infection and completed the course. Repeated bronchoscopies were required since the patient was unable to wean off the ventilator. Bronchoscopies continued to show thick secretions, but no source of respiratory infection was identified.

Endocrinology was consulted due to a new pituitary macroadenoma being seen on CT and MRI of the brain. The patient did present with acromegaloid features, such as macroglossia, coarse facial features, and large hands. She was sedated with propofol and fentanyl. She also received Lantus and sliding-scale insulin for her insulin requirements.

**Figure 1:**
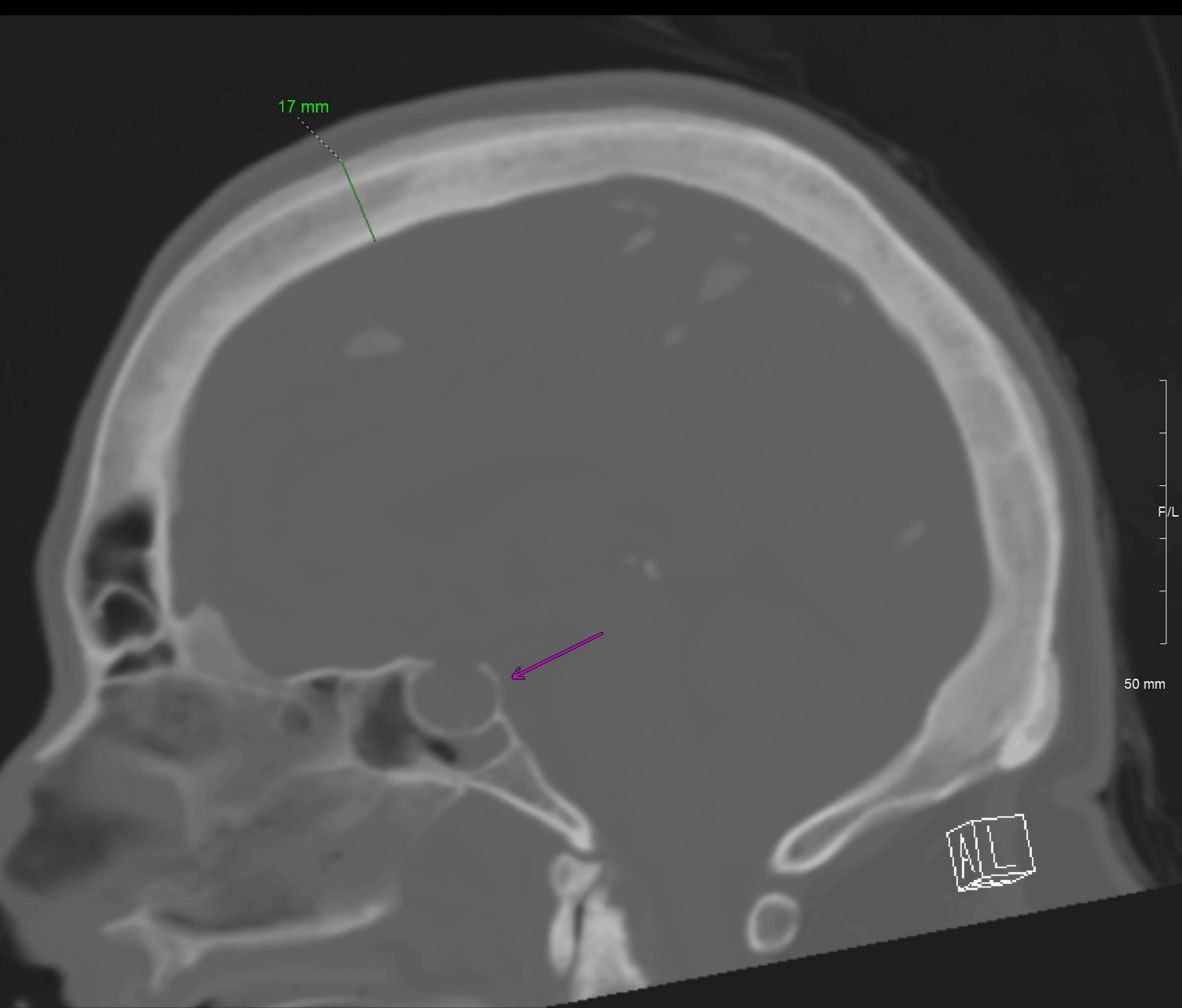
Sagittal view, right at the midline, with enlarged sella turcica (pink arrow) and maximum skull thickness of ~17 mm

**Figure 2:**
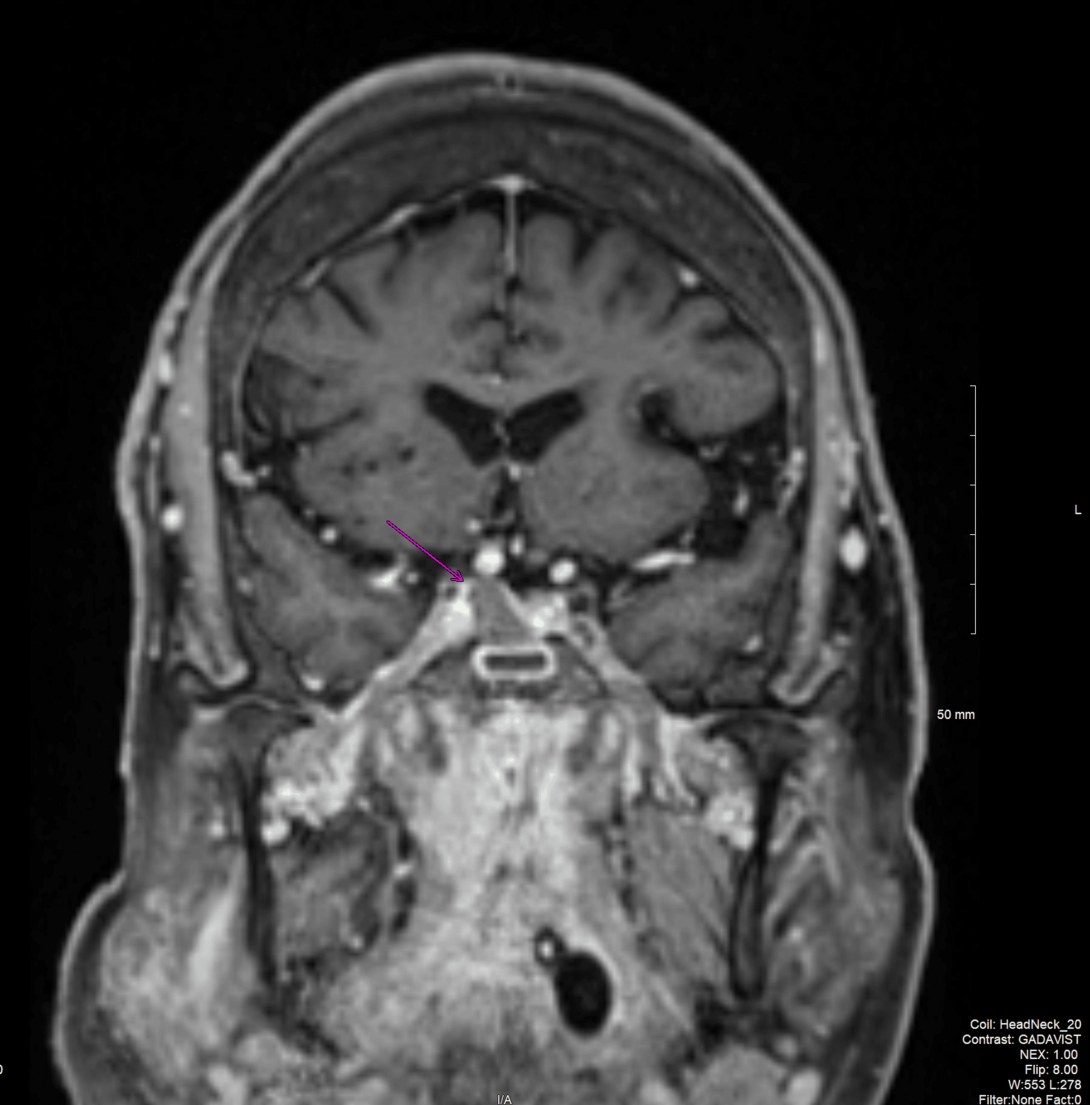
Coronal CT showing enlarged sella with asymmetrical density on the right side (pink arrow), which may represent a pituitary adenoma

**Figure 3:**
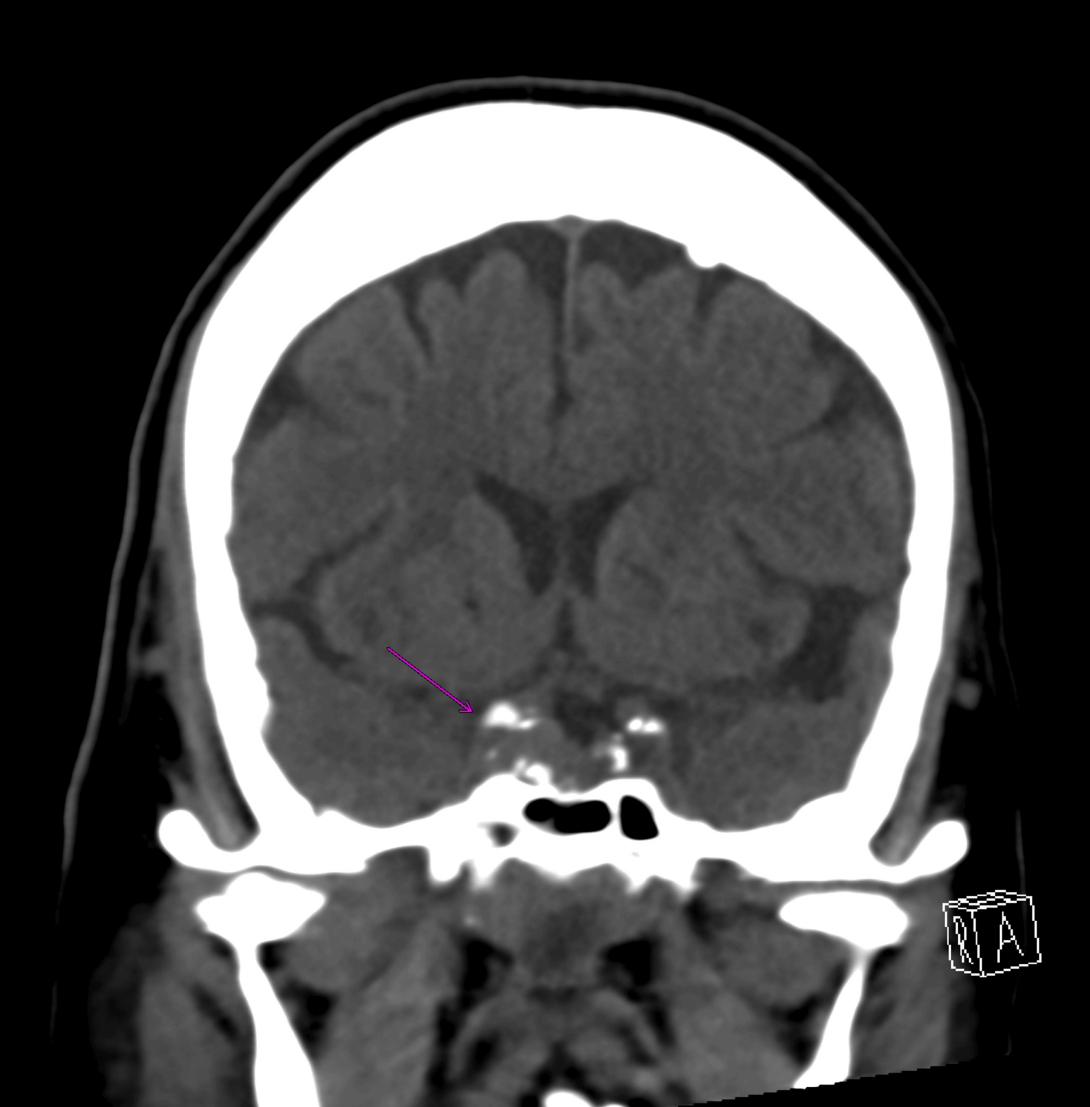
T1 fat-saturation post-contrast MRI demonstrating a T1 isointense, hypoenhancing lesion (pink arrow) in the right pituitary gland, which may represent a pituitary macroadenoma

Prior to her ICU admission, the patient had no diagnostic workup for acromegaly. Endocrinology was consulted due to the newly found pituitary adenoma seen on brain imaging. She also had significantly increased skull cortical thickness, seen on MRI (Figures 1-3). She had previously been diagnosed with hypertension, hyperlipidemia, and type 2 diabetes mellitus, for which she was already being treated with a beta blocker and insulin prior to admission. IGF-1 resulted at 570 ng/mL, and IGF-binding protein 3 (IGFBP-3) resulted at 9 µg/mL, both of which were elevated. Her thyroid-stimulating hormone (TSH) and prolactin were both mildly decreased (Table 1).

**Table 1:** Lab values for cortisol, LH, free thyroxine, TSH, FSH, and prolactin TSH, Thyroid-Stimulating Hormone; LH, Luteinizing Hormone; FSH, Follicle-Stimulating Hormone

Test Name	Test Result	Normal Reference Range
Cortisol	10.9 mcg/dL	3.4-22.5 mcg/dL
LH	3.50 mIU/mL	0.0142-0.0523 mIU/mL (postmenopausal)
Free Thyroxine	1.3 ng/dL	0.9-1.8 ng/dL
3rd Generation TSH	0.434 mcIU/mL	0.550-4.780 mcIU/mL
FSH	17.0 mIU/mL	1.5-33.4 mIU/mL
Prolactin	2.1 ng/mL	2.8-29.2 ng/mL

**Figure 4:**
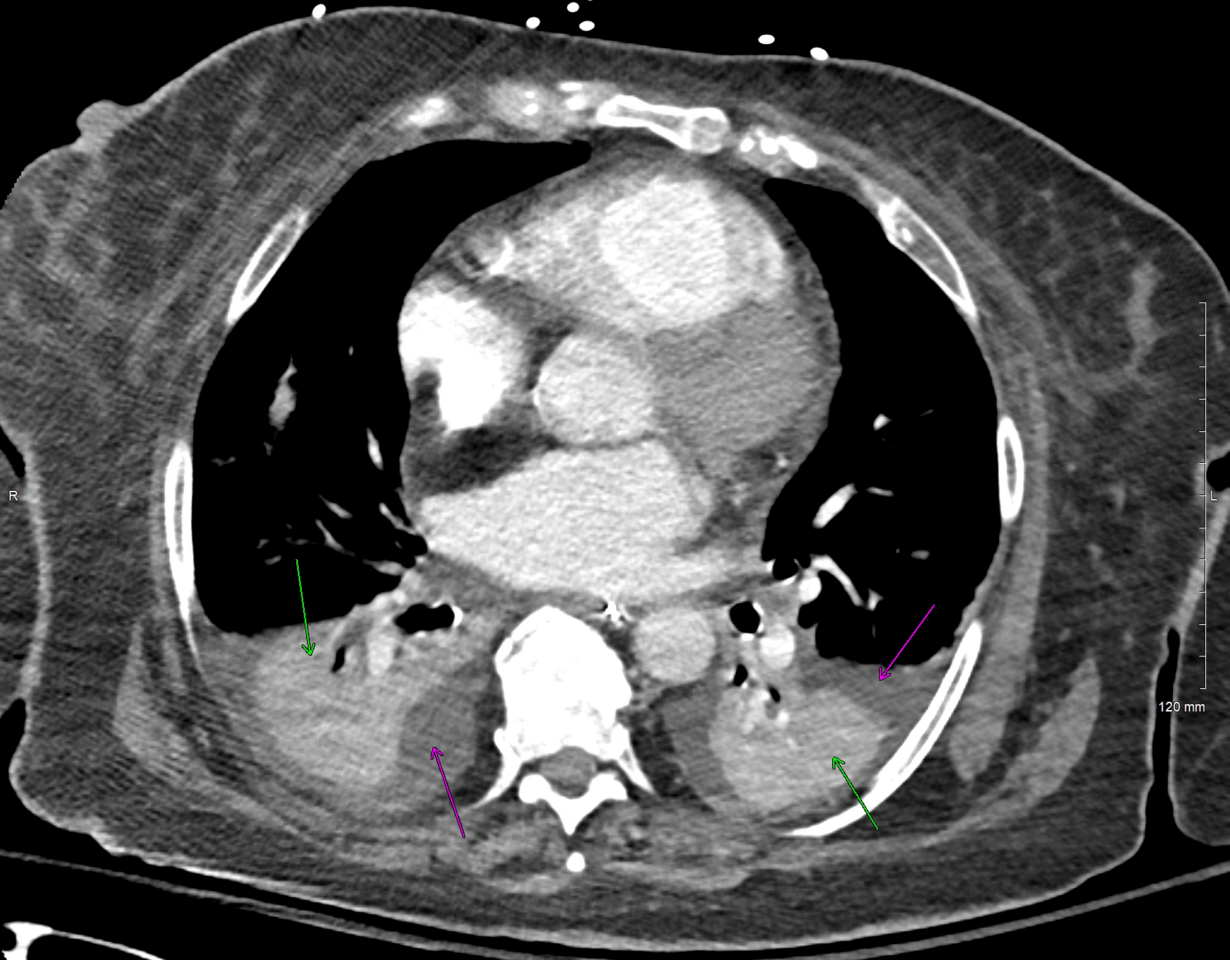
CT chest, axial slice, soft tissue window, showing bilateral pleural effusions (pink arrows), with associated complete collapse of the left lower lobe and near-complete collapse of the right lower lobe (green arrows)

It is important to reiterate that her primary cause of admission was acute hypoxic failure secondary to seizure and cardiac arrest, with concern for non-ST-elevation myocardial infarction (NSTEMI), not her incidental findings of acromegaly. Transthoracic echocardiogram (TTE) showed an ejection fraction (EF) of 75%, with mild left ventricular hypertrophy, no regional wall motion abnormalities, and indeterminate diastolic function. On repeat TTE, her EF decreased to 55% within a two-week duration. Neurosurgery was consulted due to the new pituitary mass and seizure, and they then recommended starting prophylactic lacosamide and electroencephalogram (EEG), which was unremarkable. CT of the chest showed a bilateral pleural effusion with bilateral lung collapses (Figure 4). During her bronchoscopies, the patient was noted to have excessive dynamic airway collapse (EDAC), as well as thick secretions, which may have contributed to her difficulty in ultimately weaning off the ventilator. 

Given that the patient was suffering from more pressing issues in the ICU, we hypothesized that she had undiagnosed acromegaly based on the MRI findings, as well as the IGF-1 and IGFBP-3 values. As for her treatment course, the patient received insulin for type 2 diabetes mellitus. Bronchoscopies were performed to help assess the cause of acute hypoxic respiratory failure. She was placed on hydrocortisone for stress dosing of steroids at a dose of 100 mg q12, which was eventually titrated down. She was placed on prophylactic anti-epileptics due to concern for seizures. No acute intervention was made regarding her pituitary adenoma.

After being in the ICU for 14 days, the family of the patient decided to pursue comfort care measures, resulting in her death.

## Discussion

The symptoms of acromegaly are not always obvious, which poses large challenges to diagnosis. Vague symptoms, such as headache, shortness of breath, and fatigue, may be the initial presenting signs pointing toward OSA. According to Langlois et al. (2020), OSA can worsen cardiac dysfunction, affect quality of life, and ultimately influence the mortality of patients with acromegaly [5]. The constellation of symptoms across different organ systems is crucial for early diagnosis. Delayed diagnosis has significant impacts on the quality of life and mortality that these patients face.

The respiratory and cardiac complications that acromegaly patients face are severe and not uncommon. In this case, the patient suffered recurrent mucus plugging, lung collapse, and weakness of the general respiratory tract, such that she was unable to wean off the ventilator. The most likely cause for this was the high GH/IGF-1 levels, leading to simultaneous and proliferative changes in lung musculature [5]. Since there was no clear source of infection or structural variation found in this patient that could explain her inability to wean off the ventilator, it could be proposed that her hypoxia was secondary to these changes due to IGF-1.

Cardiovascular complications play a significant role in the morbidity and mortality of acromegaly patients. As a delay in diagnosis occurs, the mortality rate of acromegaly patients increases. According to Mizera et al. (2018), secondary comorbidities, such as diabetes and hypertension, which are already elevated in this patient population, elevate this risk even further [6]. This patient did have a decline in her EF over a two-week period, with mild left ventricular hypertrophy. It seems that, in the short term, GH/IGF-1 can have some positive effects on cardiac contractility. However, in the long term, GH/IGF-1 can lead to diastolic dysfunction, systolic dysfunction, and eventually full-blown cardiomyopathy [2]. For this patient, the ultimate cause of her sudden cardiac arrest was still not known at the time of her death.

In a cohort study in Sweden, conducted by Esposito et al. (2020), the average delay in diagnosis was 5.5 years, with many patients experiencing a 10-year delay. A longer delay in diagnosis was associated with a higher number of complications, and women had a longer delay compared to men. Patients with more comorbidities also had a longer delay in diagnosis compared to those without [7]. As established by the Swedish study, the diagnosis of acromegaly is complex and challenging, but crucial for the prevention of life-threatening complications later in life.

## Conclusions

In summary, acromegaly is an incredibly complex disease process, with not just endocrine manifestations, but cardiac and respiratory sequelae as well. Diagnosis is made based on elevated levels of IGF-1 and/or an oral glucose tolerance test (OGTT). High levels of GH/IGF-1 have a simultaneous proliferative effect on lung growth and a degenerative effect on smooth muscle, leading to a mixed obstructive and restrictive lung disease. Arterial hypertension shapes cardiac remodeling, with diastolic blood pressure being the most predictive indicator for hypertrophy, as well as cardiac mortality, in acromegaly. Acromegaly is associated with increased morbidity and mortality, especially in undiagnosed and untreated patients. Delays in the diagnosis of acromegaly can cause life-threatening respiratory and cardiac complications, leading to increased morbidity and mortality, as seen in the case of this patient. In conclusion, the diagnosis of acromegaly is challenging, but crucial to the prevention of life-threatening complications later in life.
